# Supplementary material for: The role of absorption in 3D electron diffraction dynamical structure refinement
Source: Acta Crystallogr A Found Adv. 2026 Sep 21;82(Pt 6):409–18. doi: 10.1107/S2053273326009459 (PMC13720707; doi:10.1107/S2053273326009459)
Supplement: Supplementary file 1 [file a-82-00409-sup1.pdf]

Acta Cryst A

FOUNDATIONS
ADVANCES

Volume 82 (2026)

Supporting information for article:

The role of absorption in 3D electron diffraction dynamical structure refinement

Benjamin Colmey, Tiarnan A.S. Doherty, Shreshth A. Malik and Paul A. Midgley

Supplementary Information: The role of absorption in dynamical three-dimensional electron-diffraction structure refinement

Benjamin Colmey[a], Tiarnan A. S. Doherty[a,b], Shreshth A. Malik[b], and Paul A. Midgley[a]

[a]Department of Materials Science and Metallurgy, University of Cambridge, 27 Charles Babbage Rd, Cambridge, CB3 0FS, UK

[b]OATML, Department of Computer Science, University of Oxford, Wolfson Building, Parks Rd, Oxford, OX1 3QD, UK

Overview

This Supplementary Information provides additional figures, derivations, simulation parameters and results supporting the main manuscript.

1 Derivation of the Two-Beam Integrated Intensity in the Presence of Absorption for Centrosymmetric Crystals

Beginning from the modified two-beam expression for the diffracted-beam intensity under absorption:

$$|\,\psi_g\,|^2_{\mathrm{abs}} = e^{-2\kappa_0 t}\,\frac{1}{2}\left[\frac{\cosh(2\Delta\kappa\,t)}{1+x^2} - \frac{\cos(2\pi\Delta k\,t)}{1+x^2}\right]. \tag{S1}$$

Using the identities

$$\cosh 2\theta = 1 + 2\sinh^2\theta, \qquad \cos 2\theta = 1 - 2\sin^2\theta, \tag{S2}$$

we obtain

$$|\,\psi_g\,|^2_{\mathrm{abs}} = e^{-2\kappa_0 t}\left[\frac{\sinh^2(\Delta\kappa t)}{1+x^2} + \frac{\sin^2(\pi\Delta k t)}{1+x^2}\right], \tag{S3}$$

where

$$\Delta\kappa = \frac{\pi}{\xi_g'\sqrt{1+x^2}}, \qquad \Delta k = \frac{\sqrt{1+x^2}}{\xi_g}, \qquad x = S_g\xi_g. \tag{S4}$$

Defining

$$A = \frac{\pi t}{\xi_g}, \qquad B = \frac{\pi t}{\xi_g'}, \tag{S5}$$

the two terms in Eq. S3 may be written as

$$\frac{\sin^2(\pi\Delta k\, t)}{1+x^2} = \frac{\sin^2\left(A\sqrt{1+x^2}\right)}{1+x^2}, \tag{S6}$$

and

$$\frac{\sinh^2(\Delta\kappa\, t)}{1+x^2} = \frac{\sinh^2\left(B(1+x^2)^{-1/2}\right)}{1+x^2}. \tag{S7}$$

Thus the absorbed integrated intensity is

$$I_{\text{abs}}^{\text{int}}(t) = \int_{-\infty}^{\infty} |\psi_g|_{\text{abs}}^2\, dx = e^{-2\kappa_0 t}\left\{(1)+(2)\right\}, \qquad dx = \xi_g\, dS_g. \tag{S8}$$

$$(1) = \int_{-\infty}^{\infty} \frac{\sin^2\left(A\sqrt{1+x^2}\right)}{1+x^2}\, dx = \pi \int_0^A J_0(2u)\, du. \tag{S9}$$

where J_0 is the zeroth order Bessel function of the first kind.

For small u, $J_0(2u)$ can be Taylor expanded:

$$J_0(2u) = 1 - u^2 + \frac{u^4}{4} + \cdots, \tag{S10}$$

so that

$$(1) = \pi\left(A - \frac{A^3}{3} + \frac{A^5}{20} + \cdots\right). \tag{S11}$$

Substituting $A = \pi t/\xi_g$, this becomes

$$(1) = \frac{\pi^2 t}{\xi_g}\left(1 - \frac{\pi^2 t^2}{3\xi_g^2} + \frac{\pi^4 t^4}{20\xi_g^4} + \cdots\right). \tag{S12}$$

This three-term expansion remains within approximately 10% of the exact Bessel-function integral for t values up to $\xi_g/2$.

The anomalous absorption term is

$$(2) = \int_{-\infty}^{\infty} \frac{\sinh^2\left(B(1+x^2)^{-1/2}\right)}{1+x^2}\, dx. \tag{S13}$$

For $B = \pi t/\xi_g' \ll 1$,

$$\sinh^2\theta \approx \theta^2, \qquad \theta = B(1+x^2)^{-1/2}. \tag{S14}$$

Therefore

$$(2) \approx \int_{-\infty}^{\infty} \frac{B^2}{(1+x^2)^2}\, dx = B^2\frac{\pi}{2} = \frac{\pi^3 t^2}{2\xi_g'^2}. \tag{S15}$$

The non-absorbed integrated intensity is

$$I^{\mathrm{int}}_{\mathrm{no\,abs}}(t) = \int_{-\infty}^{\infty} |\psi_g|^2_{\mathrm{no\,abs}}\, dx = (1). \tag{S16}$$

Hence

$$\frac{I^{\mathrm{int}}_{\mathrm{abs}}(t)}{I^{\mathrm{int}}_{\mathrm{no\,abs}}(t)} = \frac{e^{-2\kappa_0 t}\{(1)+(2)\}}{(1)} = e^{-2\kappa_0 t}\left[1 + \frac{(2)}{(1)}\right]. \tag{S17}$$

Using Eqs. S12 and S15,

$$\frac{(2)}{(1)} \approx \frac{\dfrac{\pi^3 t^2}{2\xi_g'^2}}{\dfrac{\pi^2 t}{\xi_g}\left(1 - \dfrac{\pi^2 t^2}{3\xi_g^2} + \dfrac{\pi^4 t^4}{20\xi_g^4}\right)} = \frac{\pi t\, \xi_g}{2\xi_g'^2}\left(1 - \frac{\pi^2 t^2}{3\xi_g^2} + \frac{\pi^4 t^4}{20\xi_g^4}\right)^{-1}. \tag{S18}$$

Finally,

$$\frac{I^{\mathrm{int}}_{\mathrm{abs}}(t)}{I^{\mathrm{int}}_{\mathrm{no\,abs}}(t)} \approx e^{-2\kappa_0 t}\left[1 + \frac{\pi t\, \xi_g}{2\xi_g'^2}\left(1 - \frac{\pi^2 t^2}{3\xi_g^2} + \frac{\pi^4 t^4}{20\xi_g^4}\right)^{-1}\right]. \tag{S19}$$

To assess the validity of Eq. S19, its thickness dependence is examined alongside the ratio of intensities computed by numerical integration of Main Text Eqs. 4 and 6 in Fig. S1. Here, Fig. S1 (a) is identical to Main Text Fig. 2 (a), with the x-axis extended to 350 nm, to better observe the high-t dependence. As can be observed, the fitted values, $\bar{\lambda} = 90.7$ nm and $\sigma = 3.2$ nm, differ slightly from the values given in the Main Text of 89.9 nm and 2.0 nm, respectively, due to the extension of the x-axis.

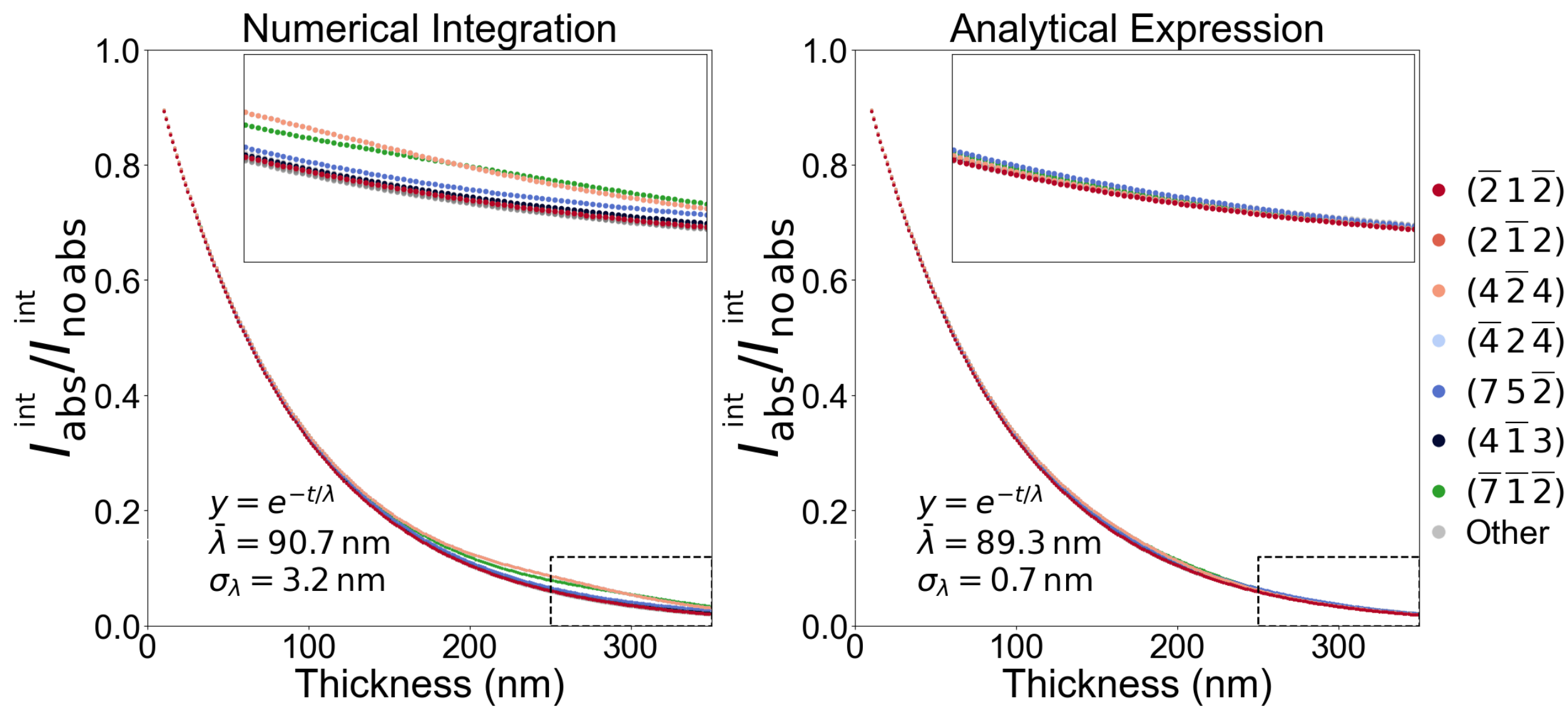

Figure S1: Ratio of absorbed to non-absorbed two-beam integrated intensities $I_{\text{abs}}^{\text{int}}/I_{\text{no abs}}^{\text{int}}$ for CsPbBr3 (orientation 1, as defined in the Main Text.) (a) Numerical integration, with an inset showing the high-thickness region. (b) Analytic approximation given by Eq. S19.

Fig. S1(b) corresponds to Eq. S19. The poorer agreement at larger thickness reflects the breakdown of the truncated Bessel-function expansion as t approaches ξ_g of the strongest reflections.

To further quantify this agreement, a residual between the numerically integrated and analytical ratios can be calculated:

$$R_1(t) = \frac{\sum_g \left| \left(\frac{I_{\text{abs},g}^{\text{int}}}{I_{\text{no abs},g}^{\text{int}}} \right)_{\text{num}} - \left(\frac{I_{\text{abs},g}^{\text{int}}}{I_{\text{no abs},g}^{\text{int}}} \right)_{\text{analytical}} \right|}{\sum_g \left(\frac{I_{\text{abs},g}^{\text{int}}}{I_{\text{no abs},g}^{\text{int}}} \right)_{\text{num}}} \tag{S20}$$

The resulting $R_1(t)$ values are shown in Fig. S2.

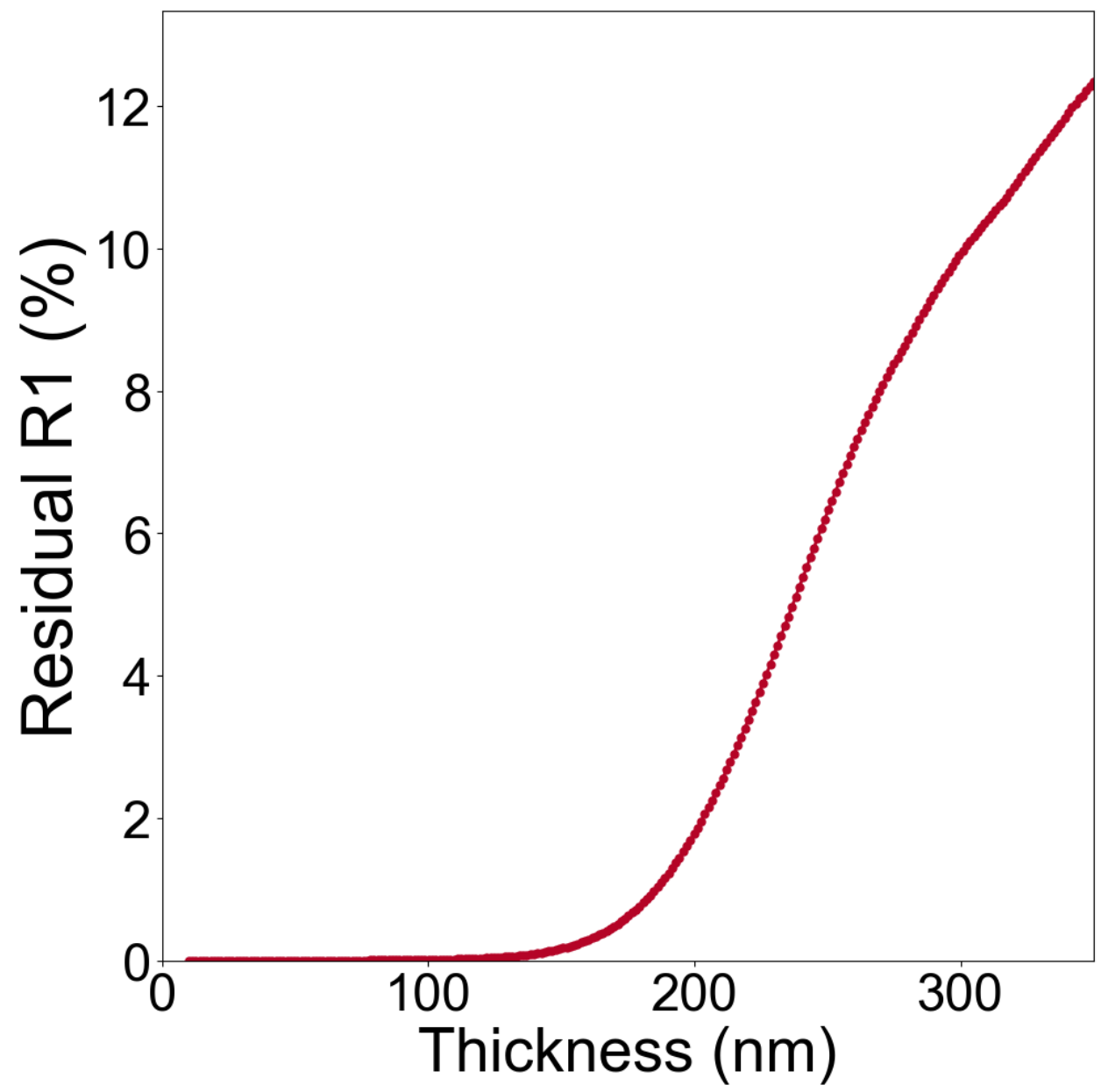

Figure S2: Residual R_1 between the numerical integration and the closed-form analytical approximation for the two-beam integrated intensity ratio, defined in Eq. S20, for the values shown in Fig. S1.

2 Extension to Non-centrosymmetric Crystals

As discussed in (Bird, 1990), in the case of non-centrosymmetric crystals, the elastic and absorptive Fourier coefficients (U_g and U'_g) may possess different phases. Following Bird, we can write:

$$U_g = |U_g|e^{i\phi_g}, \qquad U'_g = |U'_g|e^{i\phi'_g}, \qquad \Delta\phi_g = \phi_g - \phi'_g, \tag{S21}$$

with

$$U_g^{\text{tot}} = -|U_g|e^{i\phi_g} - i|U'_g|e^{i\phi'_g}. \tag{S22}$$

Defining

$$R_g = \frac{|U'_g|}{|U_g|}, \tag{S23}$$

we obtain, following Bird,

$$U_g^{\text{tot}} \simeq -\left[|U_g| + |U'_g|\sin(\Delta\phi_g)\right] e^{i\phi_g} e^{iR_g\cos(\Delta\phi_g)}. \tag{S24}$$

Taking the modulus squared and retaining terms to first order in $R_g = |U'_g|/|U_g|$, so that terms of order R_g^2 and higher are neglected, gives

$$|U_g^{\text{tot}}|^2 \simeq |U_g|^2 + 2|U_g||U'_g|\sin(\Delta\phi_g). \tag{S25}$$

For the Friedel opposite reflection,

$$U_{-g} = U_g^* = |U_g|e^{-i\phi_g}, \qquad U'_{-g} = U_g'^* = |U'_g|e^{-i\phi'_g}, \tag{S26}$$

such that

$$\Delta\phi_{-g} = -\Delta\phi_g. \tag{S27}$$

Therefore,

$$|U_{-g}^{\text{tot}}|^2 \simeq |U_g|^2 - 2|U_g||U'_g|\sin(\Delta\phi_g). \tag{S28}$$

and

$$|U_{\pm g}^{\text{tot}}|^2 \simeq |U_g|^2 \left[1 \pm 2\frac{|U'_g|}{|U_g|}\sin(\Delta\phi_g)\right]. \tag{S29}$$

Here the + and − signs correspond to the two Friedel-related reflections $\pm g$, respectively.

Using perturbation theory, Bird showed that this first-order modification to U_g produces a corresponding first-order correction to the ordinary two-beam intensity

$$I_g = \left[U_g^2 + 2U_gU'_g\sin(\Delta\phi_g)\right]\frac{\sin^2\left[\frac{\pi t}{K}(U_g^2+W^2)^{1/2}\right]}{U_g^2+W^2}. \tag{S30}$$

Or equivalently,

$$I_g = \left[1 + \frac{2U'_g}{U_g}\sin(\Delta\phi_g)\right]\frac{\sin^2\left[\frac{\pi t}{K}(U_g^2+W^2)^{1/2}\right]}{1+\frac{W^2}{U_g^2}}. \tag{S31}$$

This expression differs slightly from Eq. 26 in (Bird, 1990) because of our choice of using the crystallographic convention in defining the electron wavevector (in vacuo) as $K = 1/\lambda$; Bird uses $2\pi/\lambda$. This choice also affects other terms leading to the following changes between Bird (1990) and this paper:

$$U_g \to 4\pi^2 U_g, \qquad U'_g \to 4\pi^2 U'_g, \qquad W^2 \to (4\pi^2)^2 W^2. \tag{S32}$$

The right-hand side of Eq. S31 resembles the ordinary two-beam oscillation terms used in the main text Eq. 4. The excitation error (deviation parameter) S_g is related to W through $S_g = W/K$ and the excitation error may be defined using:

$$2KS_g = K^2 - |\mathbf{K} + \mathbf{g}|^2 \tag{S33}$$

For a reflection $\mathbf{g}$ that lies inside the Ewald sphere $S_g > 0$.

In the main text we defined the extinction distance $\xi_g = K/|U_g|$ and $x = \xi_g S_g$.

Thus $x^2 = W^2/U_g^2$.

Substituting these relations into Eq. S31, it is straightforward to show that:

$$I_g = \left[1 + 2\frac{U'_\mathbf{g}}{U_\mathbf{g}} \sin(\Delta\phi_g)\right] \frac{\sin^2\left[\frac{\pi t}{\xi_g}\sqrt{1+x^2}\right]}{1+x^2}. \tag{S34}$$

This is the ordinary two-beam intensity used in the present work (Main Text Eq. 4), with an additional first-order non-centrosymmetric absorptive correction.

For centrosymmetric crystals, $\Delta\phi_g = 0$ or π, so the first-order term proportional to $\sin(\Delta\phi_g)$ vanishes. In this sense, for centrosymmetric crystals, the first absorption correction to the diffracted intensity is second-order in $R_g = |U'_g|/|U_g|$, i.e. it arises from the R_g^2 and higher-order terms neglected in Eq. S29.

In contrast, for non-centrosymmetric crystals, $\Delta\phi_g \neq 0$ in general. The first-order term therefore survives, giving additional Friedel-pair asymmetry. Anomalous absorption is therefore expected to have a greater influence in non-centrosymmetric crystals. Additionally, since the additional first-order factor in Eq. S34 is independent of the excitation-error variable x, it survives integration over S_g directly.

Although it should in principle be possible to derive the full exact second-order absorbed two-beam solution for non-centrosymmetric crystals, such a treatment is beyond the scope of the present work. Instead, the effect may be investigated using many-beam dynamical simulations, where absorption, multiple scattering, and non-centrosymmetric diffraction effects are treated simultaneously.

2.1 Many-beam Simulations of Non-centrosymmetric Absorption Effects

While non-centrosymmetric quartz was shown to exhibit only weak absorption effects, both in simulations and in experimental refinements, the analysis in the main text suggests that this behaviour

arises primarily from its relatively light constituent scatterers ($Z_{\mathrm{Si}} = 14,\ Z_{\mathrm{O}} = 8$). It nevertheless remains unclear whether non-centrosymmetric crystals experience an additional absorption contribution relative to structurally similar centrosymmetric systems with comparable scattering strengths and thermal displacements.

To investigate this possibility, gallium arsenide (GaAs) and germanium (Ge) were selected as a non-centrosymmetric/centrosymmetric comparison pair. Both adopt tetrahedrally coordinated cubic structures with similar lattice parameters and scattering strengths, with GaAs being non-centrosymmetric ($F\bar{4}3m$; $Z_{\mathrm{Ga}} = 31,\ Z_{\mathrm{As}} = 33$) and Ge centrosymmetric ($Fd\bar{3}m$; $Z_{\mathrm{Ge}} = 32$). They provide a closely matched realistic comparison system for isolating the role of inversion symmetry. Their corresponding structural parameters are summarised in Table. S1.

To impose further similarity, identical isotropic thermal displacement parameters were assigned to all three atomic species. The common value $U_{\mathrm{iso}} = 0.007262$ Å^2 ($B = 0.5734$ Å^2) was taken from the temperature-dependent Debye–Waller factors reported for diamond-structure Ge at 280 K, derived from experimentally determined phonon densities of states (Peng *et al.*, 1996).

The similarity between GaAs and Ge is also reflected in their elastic and absorptive electron scattering factors, shown in Fig. S3.

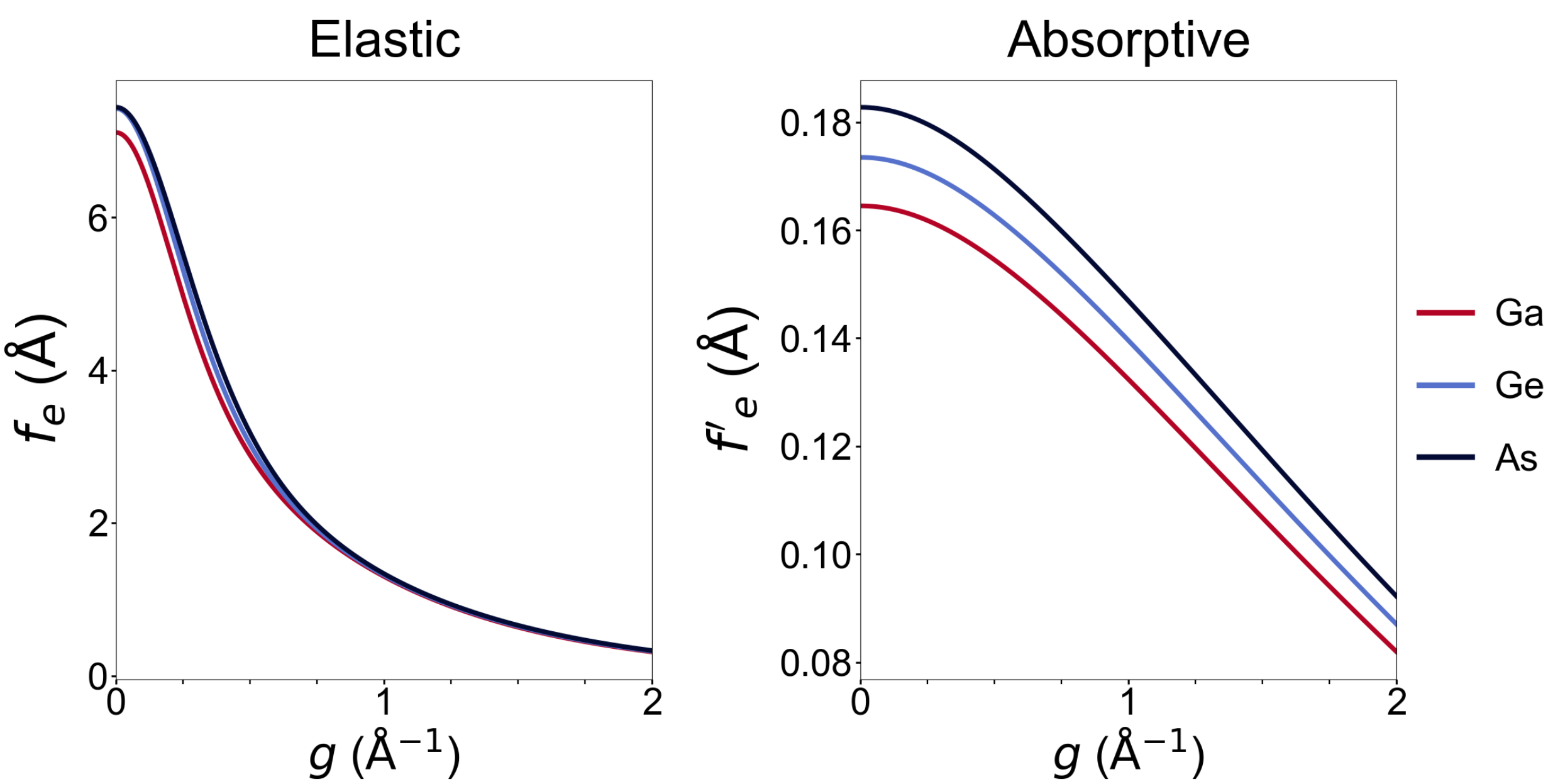

Figure S3: (a) Elastic electron scattering factors for Ga, Ge, and As using the parameterisation of Lobato *et al.* (2014). (b) Absorptive scattering factors $f'_e(s)$ calculated using the parameterisation of Thomas *et al.* (2024) at 200 keV using the common isotropic displacement parameter $U_{\mathrm{iso}} = 0.0073$ Å^2.

Using this approach, full many-beam simulations were performed with and without absorption for both structures using identical orientations and simulation parameters. Integrated intensities

computed across thickness were compared through the corresponding absorption-induced residuals $R_1(t)$ (Main Text Eq. 9).

While the earlier $CsPbBr_3$, quartz, and borane analysis could be validated directly against experiment, no equivalent experimental 3D ED dataset exists for the present centrosymmetric/non-centrosymmetric comparison. To approximate representative 3D ED conditions, the first three orientations from the $CsPbBr_3$ dataset (also shown in Main Text Fig. 3b) were used for both GaAs and Ge: $[uvw] = [-0.43, 1.00, 0.93]$, $[-0.46, 1.00, 0.89]$, and $[-0.50, 1.00, 0.85]$. These orientations were previously shown to provide a representative sampling of typical off-zone 3D ED orientations.

The resulting $R_1(t)$ curves are shown in Fig. S4, with the corresponding linear fit parameters summarised in Table S2. Both materials exhibit a broadly similar increase in residual with thickness when comparing absorptive and elastic simulated datasets. The mean fitted slope dR_1/dt is slightly larger for GaAs than for Ge (0.0294 versus 0.0271 %/nm), indicating a modest enhancement of the absorption contribution in the non-centrosymmetric case. However, the overall behaviour remains very similar between the two systems, suggesting that the conclusions of the present work extend to non-centrosymmetric crystals: while non-centrosymmetry is expected to introduce an additional absorptive contribution, its magnitude appears sufficiently small that absorption is still expected to play only a limited role under typical 3D ED conditions for materials with comparable scattering strengths.

Furthermore, while this comparison was not validated directly against experiment, the many-beam simulation framework used throughout this work has shown good overall agreement with experiment, correctly predicting *a priori* the relative magnitude of absorption effects observed across $CsPbBr_3$, quartz, and borane when accounting for both limitations of the absorptive model itself and additional sources of experimental error.

Table S1: Structure and simulation parameters used for the GaAs/Ge comparison.

	GaAs	Ge
Space group	$F\bar{4}3m$	$Fd\bar{3}m$
a, b, c (Å)	5.750, 5.750, 5.750	5.657, 5.657, 5.657
α, β, γ (°)	90, 90, 90	90, 90, 90
Cell volume (Å^3)	190.13	181.11
Accelerating voltage (keV)	200	200
Rocking-curve sampling	60	60
Integration semiangle (°)	1.5	1.5
D_{sg}	0.00	0.00
R_{sg}	0.9	0.9
g_{max} (Å^{-1})	3.3	3.3
S_g^{max} (Å^{-1})	0.03	0.03

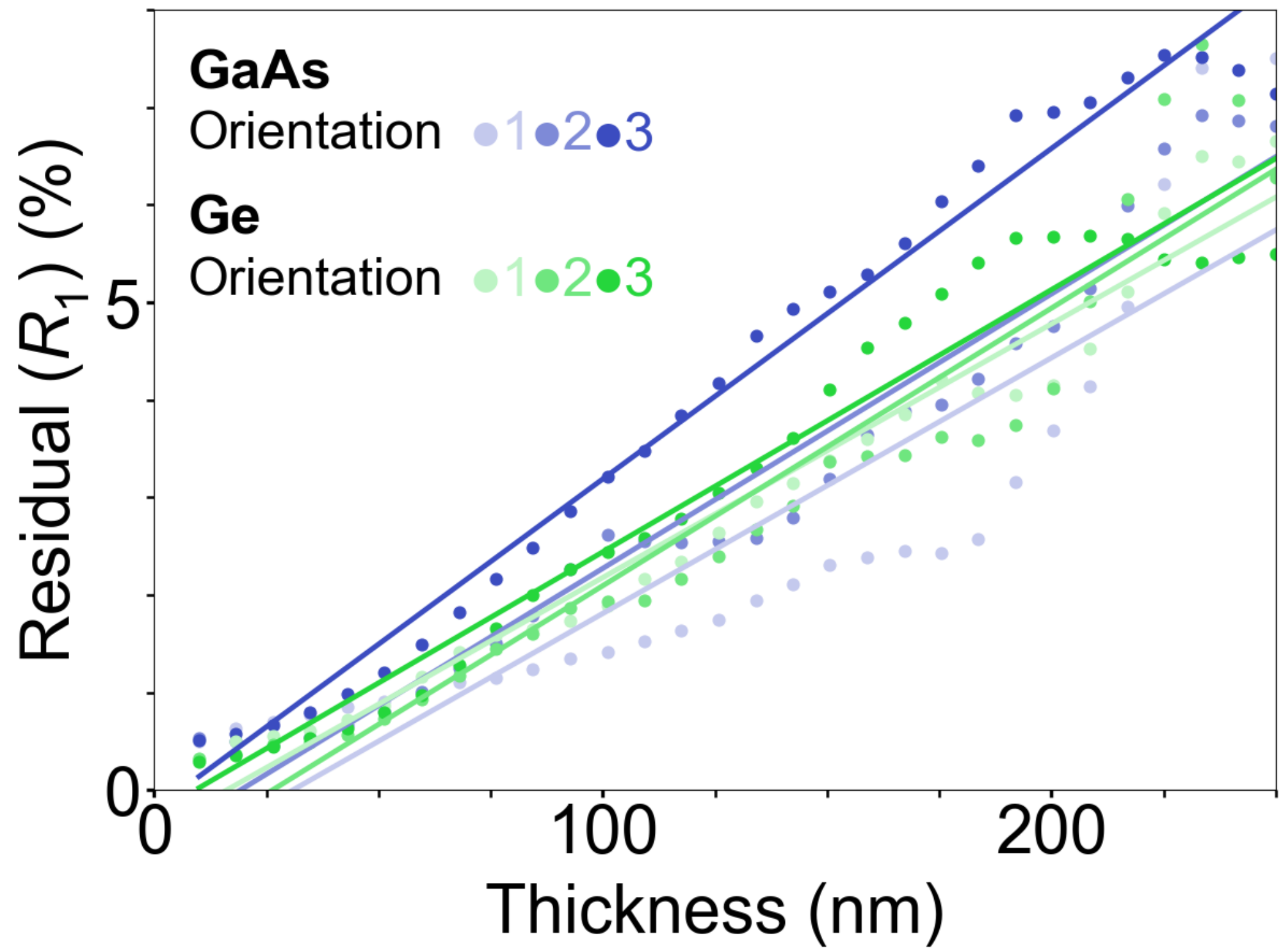

Figure S4: Integrated-intensity residual R_1 curves calculated with and without absorption for GaAs and Ge as a function of thickness. Results are shown for the three representative orientations, labelled 1, 2, 3 corresponding to $[uvw] = [-0.43, 1.00, 0.93]$, $[-0.46, 1.00, 0.89]$, and $[-0.50, 1.00, 0.85]$ respectively, using the simulation parameters summarised in Table S1. The best-fit slopes dR_1/dt across the three orientations are given in Table. S2.

Table S2: Linear $R_1(t)$ fit parameters obtained for the three representative orientations used in the GaAs/Ge comparison.

		**GaAs**		**Ge**	
**Orientation**	$[uvw]$	dR_1/dt **(%/nm)**	$R_1(t=0)$	dR_1/dt **(%/nm)**	$R_1(t=0)$
1	$[-0.43, 1.00, 0.93]$	0.0262	-0.8101	0.0260	-0.4209
2	$[-0.46, 1.00, 0.89]$	0.0281	-0.5410	0.0285	-0.7505
3	$[-0.50, 1.00, 0.85]$	0.0339	-0.1997	0.0269	-0.2468

3 Derivation of $R_1(t)$ in terms of $\bar{\lambda}$ and σ_λ

The R–factor is defined as

$$R_1(t) = \frac{\sum_i \left| \sqrt{I_i^{\text{abs}}(t)} - \sqrt{I_i^{\text{no abs}}} \right|}{\sum_i \sqrt{I_i^{\text{no abs}}}}, \tag{S35}$$

where $I_i^{\text{no abs}}$ is the intensity without absorption, and

$$I_i^{\text{abs}}(t) = I_i^{\text{no abs}}\, e^{-t/\lambda_i} \tag{S36}$$

As the mean absorption length $\bar{\lambda}$ scales all intensities equally, it does not contribute to the per–reflection variation and thus has no effect on $R(t)$. Its contribution can therefore be removed with a global scaling factor. The scaled absorbed intensity is

$$I_i^{\text{abs, scaled}}(t) = I_i^{\text{no abs}}\, \frac{e^{-t/\lambda_i}}{e^{-t/\bar{\lambda}}}, \tag{S37}$$

so that

$$\sqrt{I_i^{\text{abs, scaled}}(t)} = \sqrt{I_i^{\text{no abs}}}\, e^{-\frac{t}{2}(X_i-\mu_X)}, \qquad X_i = \frac{1}{\lambda_i},\;\; \mu_X = \frac{1}{\bar{\lambda}}. \tag{S38}$$

Substituting into (S35) gives

$$R_1(t) = \sum_i w_i \left| e^{-\frac{t}{2}(X_i-\mu_X)} - 1 \right|, \qquad w_i = \frac{\sqrt{I_i^{\text{no abs}}}}{\sum_j \sqrt{I_j^{\text{no abs}}}}. \tag{S39}$$

For small $t(X_i - \mu_X)$ the exponential can be expanded:

$$e^{-\frac{t}{2}(X_i-\mu_X)} \;\approx\; 1 - \tfrac{t}{2}(X_i - \mu_X), \tag{S40}$$

so that

$$R_1(t) \;\approx\; \tfrac{t}{2} \sum_i w_i |X_i - \mu_X|. \tag{S41}$$

The weighted variance is defined as

$$\sigma_X^2 = \sum_i w_i (X_i - \mu_X)^2, \tag{S42}$$

and the standardized variables as

$$Z_i = \frac{X_i - \mu_X}{\sigma_X}, \qquad \sum_i w_i Z_i^2 = 1, \tag{S43}$$

which yield

$$\sum_i w_i |X_i - \mu_X| = \sigma_X \sum_i w_i |Z_i|. \tag{S44}$$

If the distribution of X_i is approximately Gaussian, the standardized values behave like samples

from $Z \sim \mathcal{N}(0,1)$, for which the expectation can be found:

$$\mathbb{E}[|Z|] = \int_{-\infty}^{\infty} |z| \frac{1}{\sqrt{2\pi}} e^{-z^2/2}\, dz = \sqrt{\tfrac{2}{\pi}}. \tag{S45}$$

Hence,

$$\sum_i w_i |X_i - \mu_X| \;\approx\; \sigma_X \sqrt{\tfrac{2}{\pi}}, \qquad R_1(t) \approx \frac{t}{2}\sqrt{\tfrac{2}{\pi}}\, \sigma_X. \tag{S46}$$

The quantity σ_X may now be related to σ_λ to express the result in terms of the absorption lengths. Writing $\lambda = \bar{\lambda} + \delta\lambda$, a first–order expansion gives

$$X = \frac{1}{\lambda} \;\approx\; \frac{1}{\bar{\lambda}} - \frac{\delta\lambda}{\bar{\lambda}^2}, \qquad X - \mu_X \approx -\frac{\delta\lambda}{\bar{\lambda}^2}. \tag{S47}$$

Squaring and averaging,

$$\sigma_X^2 = \mathbb{E}\big[(X-\mu_X)^2\big] \;\approx\; \frac{1}{\bar{\lambda}^4}\, \mathbb{E}\big[(\delta\lambda)^2\big]. \tag{S48}$$

By definition,

$$\mathbb{E}\big[(\delta\lambda)^2\big] = \sigma_\lambda^2, \tag{S49}$$

and therefore

$$\sigma_X \;\approx\; \frac{\sigma_\lambda}{\bar{\lambda}^2}. \tag{S50}$$

Substituting (S50) into (S46) yields the final result:

$$R_1(t) \;\approx\; \sqrt{\tfrac{2}{\pi}}\, \frac{t\, \sigma_\lambda}{2\, \bar{\lambda}^2}. \tag{S51}$$

4 Supplementary Figures

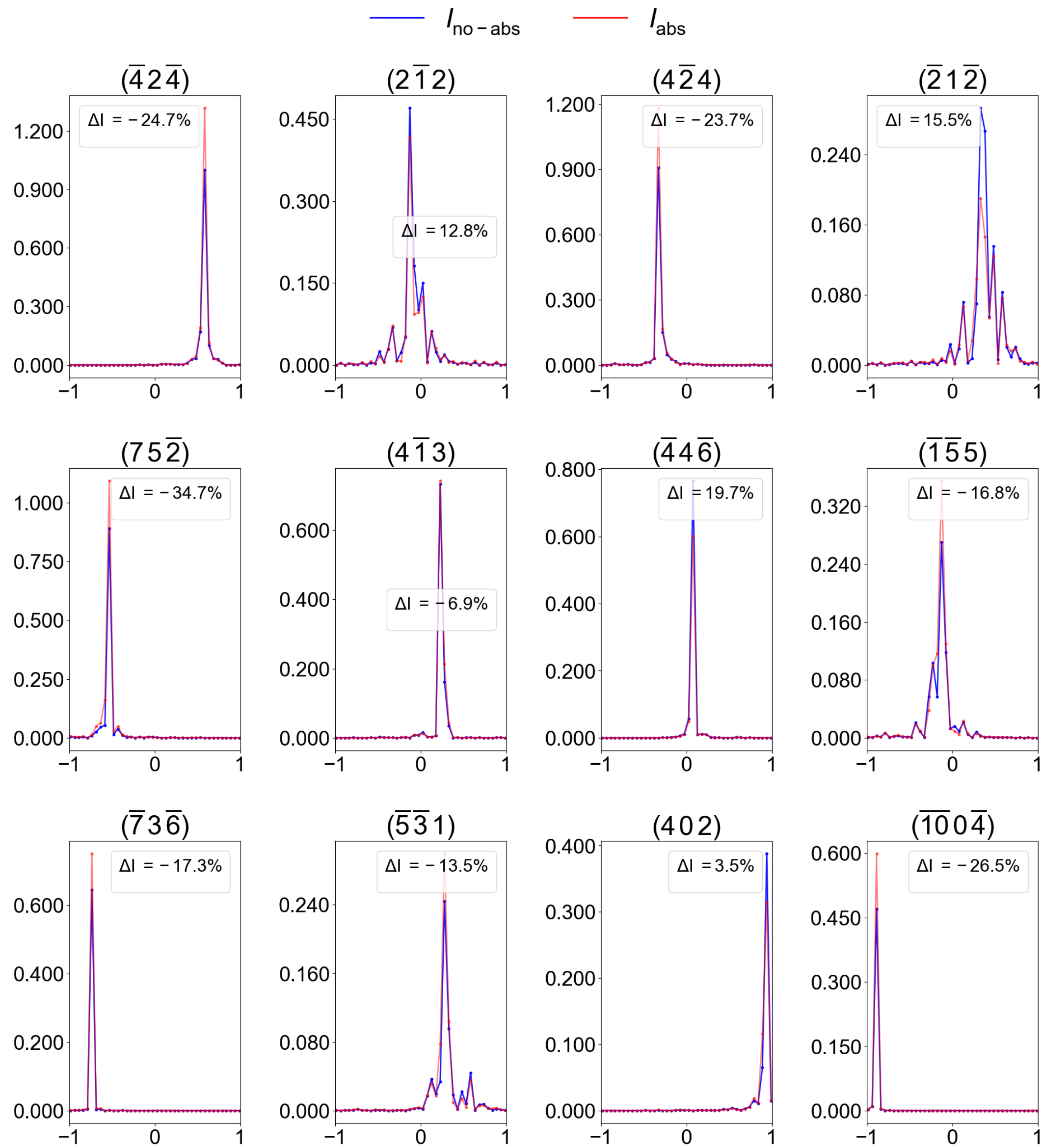

Figure S5: Many-beam rocking curves for $CsPbBr_3$ at $t = 200\,\text{nm}$, 200 keV, and $[uvw] = [-0.43, 1.00, 0.93]$ (page 1 of 3). For clarity, the x-axis is limited to $\pm 1°$, while the underlying simulation extends to $\pm 1.5°$. The absorptive curves are scaled by a physical de-attenuation factor $\exp(t/\lambda)$ with $t = 200\,\text{nm}$ and $\lambda = 88.4\,\text{nm}$ to compensate for uniform absorption due to U_0'; ΔI gives the % difference between integrated values after this correction: $\Delta I = 100 \times \frac{I_{\text{no-abs}} - I_{\text{abs}}}{I_{\text{no-abs}}}$.

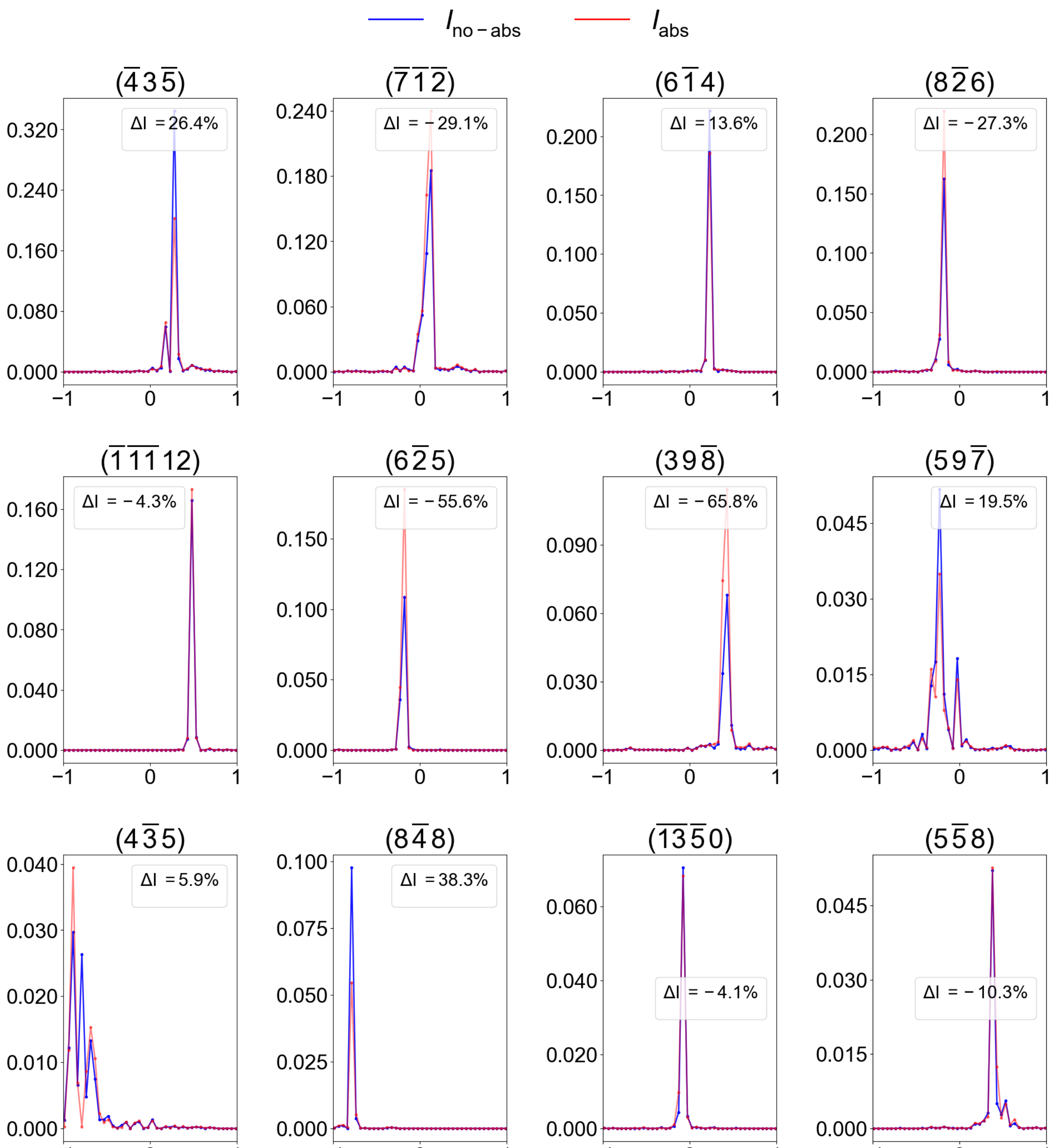

Figure S5: (continued, page 2 of 3).

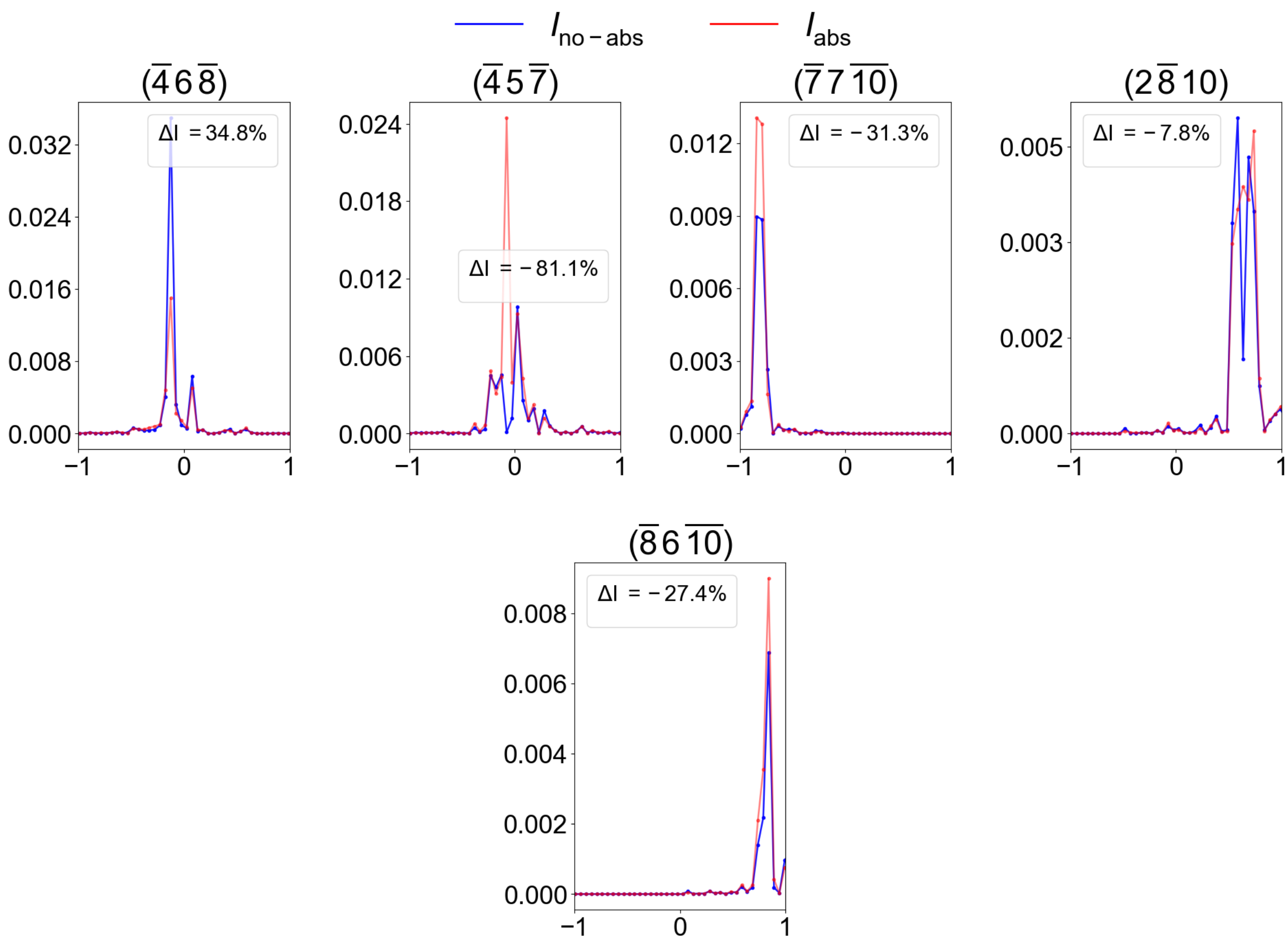

Figure S5: (continued, page 3 of 3).

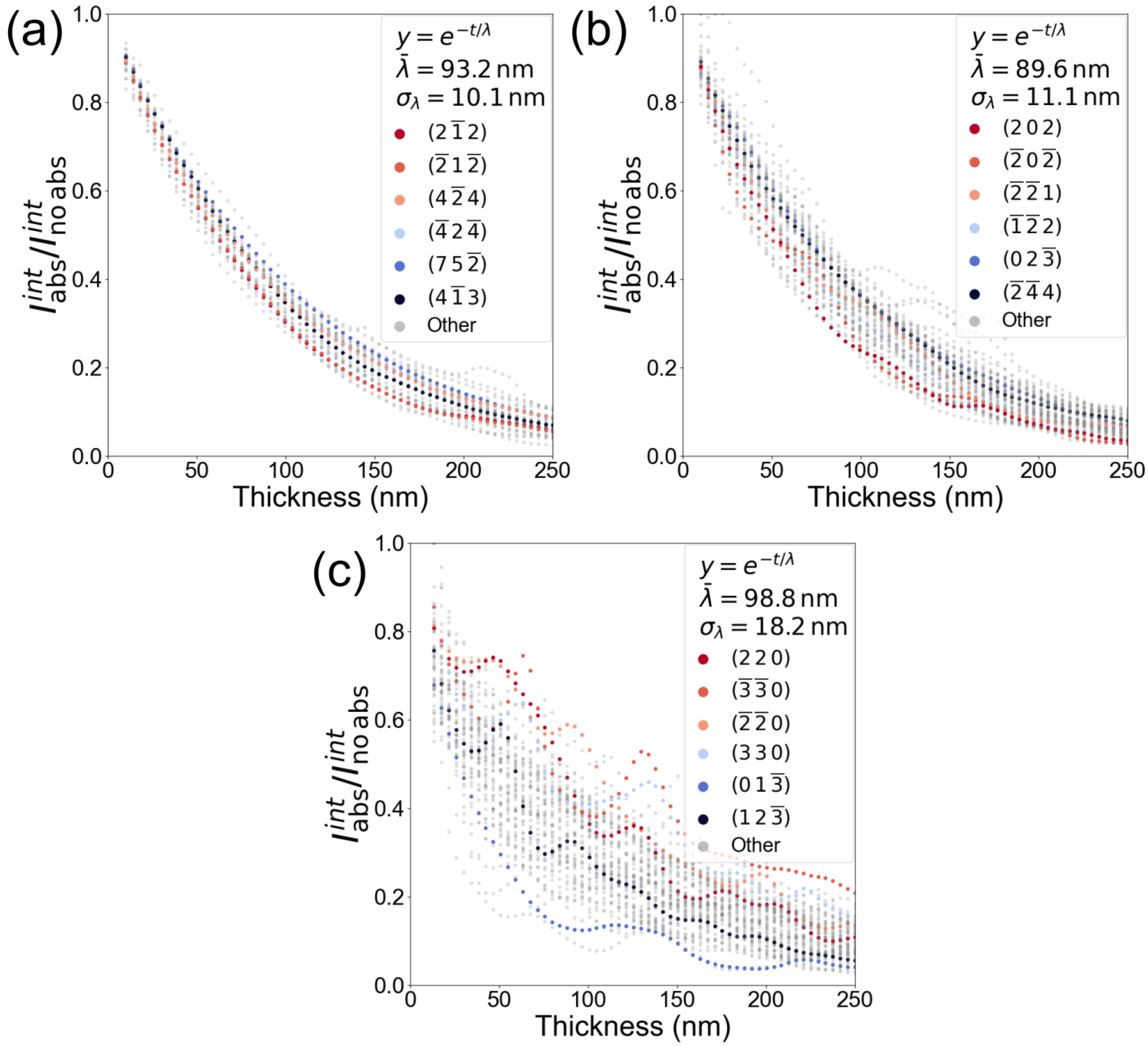

Figure S6: Orientation dependence of absorption effects in $CsPbBr_3$. (a–c) Ratio of integrated intensities $I_{\mathrm{abs}}/I_{\mathrm{no\,abs}}$ as a function of thickness from many-beam dynamical diffraction simulations for orientations 1, 8, and 19, as defined in the main text. The six most intense reflections are highlighted in colour, with all others shown in grey. Mean absorption length $\bar{\lambda}$ and standard deviation σ_λ are shown for each orientation.

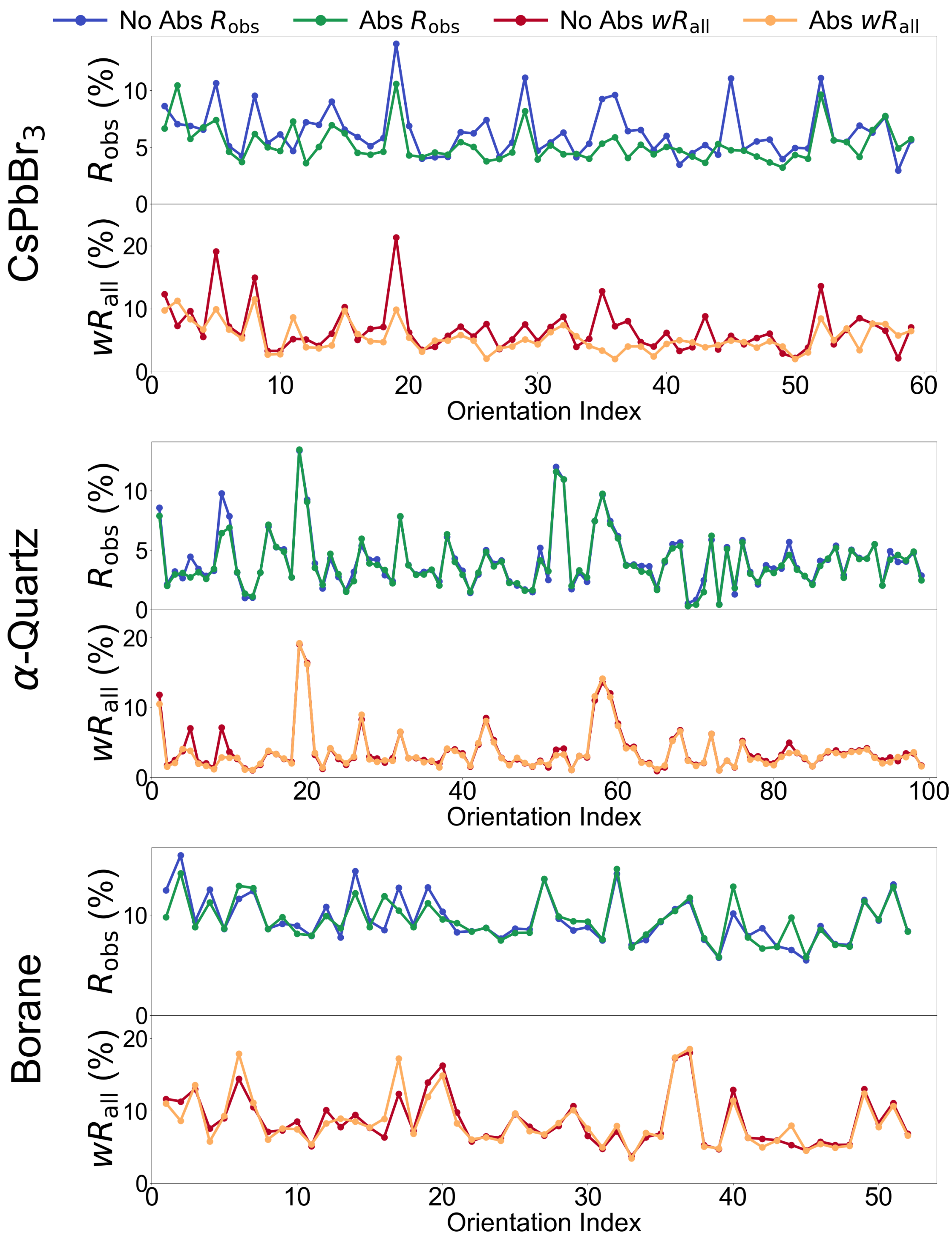

Figure S7: Results of dynamical refinements performed with and without absorption for $CsPbBr_3$, α-quartz, and borane. Residuals R_{obs} and wR_{all} are shown across all rotation indices for refinements carried out on identical datasets and parameters.

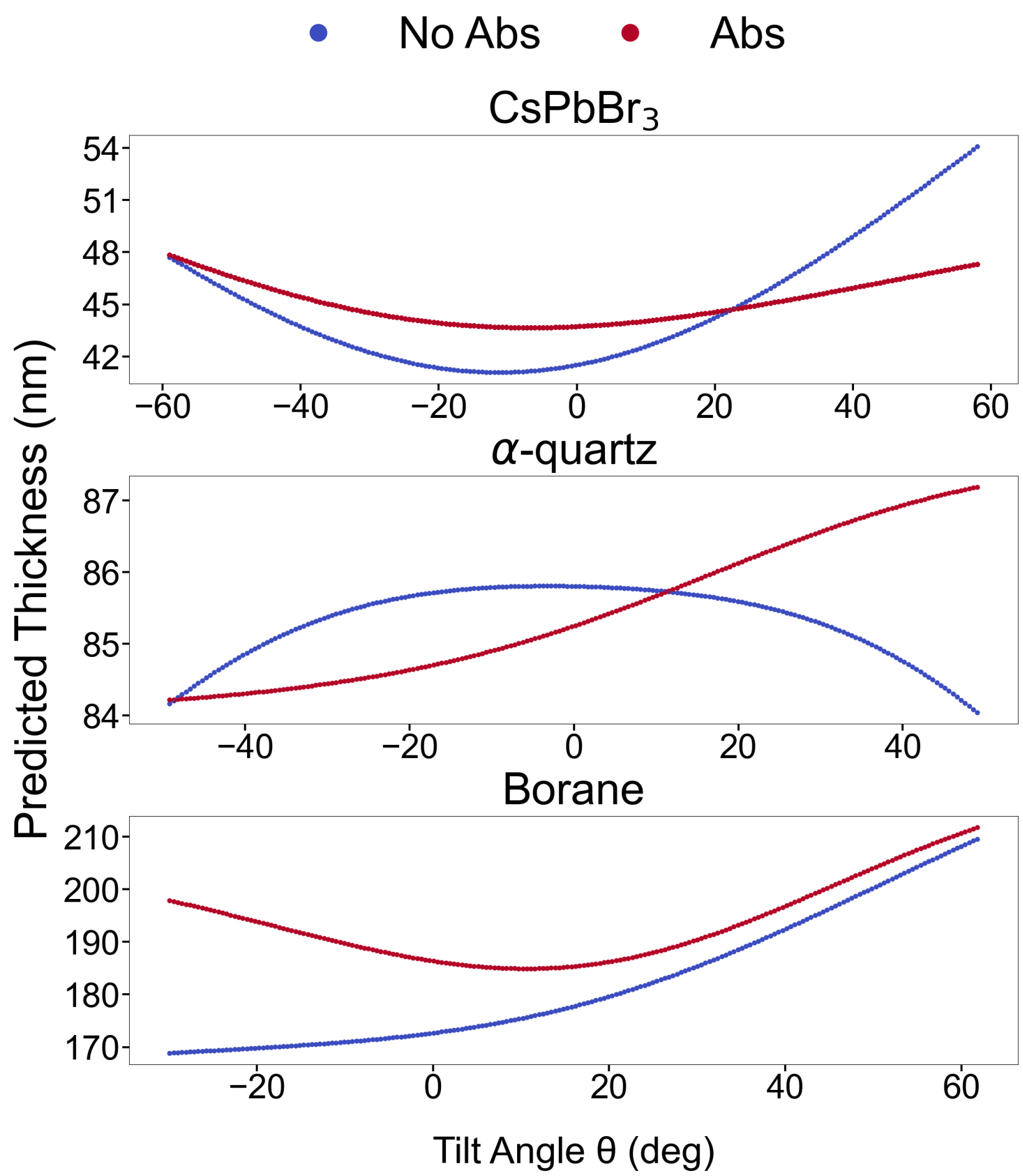

Figure S8: Experimental thickness curves recovered from dynamical refinements performed with and without absorption for $CsPbBr_3$, α-quartz, and borane datasets.

157 # 5 Supplementary Tables

Table S3: Summary of Main Text Fig. 3 (b): material dependence of absorption effects, showing slopes of $R_1(t)$ (%/nm) for representative orientations. Mean atomic number $\langle Z \rangle$, reported thickness t_{obs}, and the predicted contribution of absorption to R_{obs} are also listed.

**Material**	$\langle Z \rangle$	t_{obs} (nm)	**Orientations [uvw]**	dR_1/dt **(%/nm)**	**Pred. (%)**
**$CsPbBr_3$**	48.4	48.0	[-0.43, 1.00, 0.93]	0.0480	2.11
			[-0.46, 1.00, 0.89]	0.0418	1.83
			[-0.50, 1.00, 0.85]	0.0405	1.78
**α-Quartz**	10.0	85.0	[-1.00, 0.08, -0.28]	0.0064	0.74
			[-1.00, 0.10, -0.29]	0.0058	0.65
			[-1.00, 0.13, -0.29]	0.0046	0.52
**Borane**	2.8	170.0	[1.00, -0.49, -0.52]	0.0004	0.12
			[1.00, -0.46, -0.54]	0.0009	0.20
			[1.00, -0.43, -0.57]	0.0007	0.17

Table S4: Refinement parameters and residuals for $CsPbBr_3$, α-quartz, and borane. All settings are identical between elastic-only and absorptive refinements. Geometric filtering parameters RSg and DSg follow the conventions of (Klar et al., 2023). Values denoted with (SFs) refer to the maximum resolution of **g** vectors included in structure factor calculations.

**Parameter**	**$CsPbBr_3$**	**α-quartz**	**Borane**
Integration semiangle (°)	1.5	1.0	1.35
Rocking-curve sampling	60	42	125
D_{sg}	0.0015	0.0015	0.00
R_{sg}	0.66	0.90	0.66
g_{max} (SFs) Å^{-1}	2.5	2.5	2.5
$g_{\mathrm{max}}^{\mathrm{refine}}$ Å^{-1}	2.0	1.6	1.7
$S_g$max (SFs) Å^{-1}	0.02	0.01	0.01
**Elastic-only refinement**			
R_{obs} (%)	6.40	4.12	9.54
wR_{all} (%)	6.73	3.84	8.56
**Absorptive refinement**			
R_{obs} (%)	5.26	4.00	9.48
wR_{all} (%)	5.31	3.66	8.51

Table S5: Fractional atomic coordinates and anisotropic displacement parameters from dynamical refinements of $CsPbBr_3$ performed with and without inclusion of absorption.

	**Without Absorption**						**With Absorption**					
Atom	x	y	z	U_{11}	U_{22}	U_{33}	x	y	z	U_{11}	U_{22}	U_{33}
Cs	0.514	0.553	0.750	0.045	0.039	0.028	0.514	0.554	0.750	0.044	0.042	0.030
Pb	0.500	0.000	0.500	0.016	0.014	0.014	0.500	0.000	0.500	0.016	0.014	0.014
Br1	0.301	0.701	0.468	0.023	0.023	0.035	0.301	0.701	0.468	0.024	0.022	0.036
Br2	0.439	0.995	0.750	0.043	0.044	0.012	0.440	0.995	0.750	0.046	0.046	0.011

Table S6: Fractional atomic coordinates and anisotropic displacement parameters from dynamical refinements of quartz with and without absorption.

Atom	**Without Absorption**						**With Absorption**					
	x	y	z	U_{11}	U_{22}	U_{33}	x	y	z	U_{11}	U_{22}	U_{33}
Si	1.0000	0.4712	0.8333	0.0105	0.0109	0.0049	1.0000	0.4712	0.8333	0.0108	0.0112	0.0052
O	0.8549	0.5857	0.6189	0.0105	0.0194	0.0161	0.8548	0.5859	0.6189	0.0113	0.0197	0.0160

Table S7: Fractional atomic coordinates and anisotropic displacement parameters from dynamical refinements of borane with and without absorption.

Atom	**Without Absorption**						**With Absorption**					
	x	y	z	U_{11}	U_{22}	U_{33}	x	y	z	U_{11}	U_{22}	U_{33}
H1	0.4255	0.4902	0.2342	0.0226	0.0140	0.0596	0.4256	0.4901	0.2341	0.0230	0.0150	0.0611
H2	0.5413	0.5880	0.1026	0.1359	0.0533	0.0036	0.5411	0.5881	0.1026	0.1460	0.0660	0.0098
H3	0.7160	0.6490	0.3212	0.0481	0.0250	0.0738	0.7158	0.6493	0.3211	0.0475	0.0325	0.0782
H4	0.6173	0.2361	0.3329	0.0767	0.0176	0.0479	0.6166	0.2366	0.3331	0.0676	0.0175	0.0505
H5	0.7863	0.4412	0.4552	0.0469	0.0675	0.0335	0.7856	0.4410	0.4551	0.0496	0.0824	0.0261
H6	0.5307	0.6140	0.2931	0.0185	0.0197	0.0062	0.5314	0.6137	0.2935	0.0214	0.0166	0.0072
H7	0.4569	0.3396	0.1401	0.0437	0.0477	0.0153	0.4571	0.3400	0.1405	0.0331	0.0539	0.0124
H8	0.7439	0.4082	0.1791	0.0593	0.0791	0.0333	0.7440	0.4083	0.1789	0.0559	0.0713	0.0322
H9	0.3542	0.4459	0.3981	0.0310	0.0063	0.0960	0.3542	0.4462	0.3987	0.0331	0.0105	0.0983
H10	0.5886	0.3199	0.5888	0.0551	0.0703	0.1194	0.5884	0.3201	0.5884	0.0434	0.0709	0.1046
H11	0.3605	0.2708	0.4084	0.0298	0.0393	0.1378	0.3608	0.2705	0.4082	0.0386	0.0354	0.1238
B1	0.4328	0.3541	0.3945	0.0012	0.0047	0.0147	0.4327	0.3541	0.3946	0.0015	0.0063	0.0147
B2	0.6797	0.4485	0.4129	0.0034	0.0083	0.0051	0.6796	0.4481	0.4130	0.0033	0.0082	0.0053
B3	0.5446	0.5353	0.2009	0.0072	0.0084	0.0147	0.5447	0.5352	0.2008	0.0049	0.0093	0.0158
B4	0.5891	0.3358	0.3469	0.0033	0.0005	0.0166	0.5890	0.3358	0.3472	0.0034	0.0009	0.0152
B5	0.6543	0.4398	0.2460	0.0078	0.0081	0.0075	0.6541	0.4396	0.2460	0.0072	0.0083	0.0089
B6	0.5582	0.3828	0.5000	0.0039	0.0054	0.0030	0.5581	0.3825	0.4999	0.0041	0.0049	0.0026
B7	0.6432	0.5702	0.3274	0.0016	0.0002	0.0199	0.6430	0.5702	0.3275	0.0014	0.0009	0.0203
B8	0.4953	0.3932	0.2292	0.0112	0.0082	0.0029	0.4955	0.3933	0.2293	0.0081	0.0064	0.0079
B9	0.5755	0.5322	0.4937	0.0005	0.0004	0.0127	0.5756	0.5325	0.4941	0.0005	0.0003	0.0132
